# Cost−utility analysis of shockwave lithotripsy vs ureteroscopic stone treatment in adults

**DOI:** 10.1111/bju.15862

**Published:** 2022-08-16

**Authors:** Mary M. Kilonzo, Ranan Dasgupta, Ruth Thomas, Lorna Aucott, Sara MacLennan, Thomas Boon L. Lam, Ken Anson, Sarah Cameron, Kath Starr, Neil Burgess, Francis Xavier Keeley, Charles T. Clark, James N'Dow, Graeme MacLennan, Sam McClinton

**Affiliations:** ^1^ Health Economics Research Unit University of Aberdeen Aberdeen UK; ^2^ Department of Urology Imperial College Healthcare NHS Trust London UK; ^3^ Centre for Healthcare Randomised Trials University of Aberdeen Aberdeen UK; ^4^ Health Services Research Unit University of Aberdeen Aberdeen UK; ^5^ Academic Urology Unit University of Aberdeen Aberdeen UK; ^6^ NHS Grampian, Department of Urology Aberdeen Royal Infirmary Aberdeen UK; ^7^ Department of Urology St Georges University Hospitals NHS Foundation Trust London UK; ^8^ Warwick Clinical Trials Unit University of Warwick Warwick UK; ^9^ Department of Urology Norfolk and Norwich University Hospitals NHS Foundation Trust Norwich UK; ^10^ Bristol Urological Institute Bristol UK; ^11^ BAUS Section of Endourology Consumer/Patient Advisory Group London UK

**Keywords:** cost‐effectiveness, ureteric stones, URS, ESWL, economic evaluation

## Abstract

**Objectives:**

To assess the cost‐effectiveness, resource use implications, quality‐adjusted life‐years (QALYs) and cost per QALY of care pathways starting with either extracorporeal shockwave lithotripsy (SWL) or with ureteroscopic retrieval (ureteroscopy [URS]) for the management of ureteric stones.

**Patients and Methods:**

Data on quality of life and resource use for 613 patients, collected prospectively in the Therapeutic Interventions for Stones of the Ureter (TISU) randomized controlled trial (ISRCTN 92289221), were used to assess the cost‐effectiveness of two care pathways, SWL and URS. A health provider (UK National Health Service) perspective was adopted to estimate the costs of the interventions and subsequent resource use. Quality‐of‐life data were calculated using a generic instrument, the EuroQol EQ‐5D‐3L. Results are expressed as incremental cost‐effectiveness ratios and cost‐effectiveness acceptability curves.

**Results:**

The mean QALY difference (SWL vs URS) was −0.021 (95% confidence interval [CI] −0.033 to −0.010) and the mean cost difference was −£809 (95% CI −£1061 to −£551). The QALY difference translated into approximately 10 more healthy days over the 6‐month period for the patients on the URS care pathway. The probabaility that SWL is cost‐effective is 79% at a society's willingness to pay (WTP) threshold for 1 QALY of £30,000 and 98% at a WTP threshold of £20,000.

**Conclusion:**

The SWL pathway results in lower QALYs than URS but costs less. The incremental cost per QALY is £39 118 cost saving per QALY lost, with a 79% probability that SWL would be considered cost‐effective at a WTP threshold for 1 QALY of £30 000 and 98% at a WTP threshold of £20 000. Decision‐makers need to determine if costs saved justify the loss in QALYs.

## Introduction

The healthcare burden of urinary tract stone disease is rising, with an estimated lifetime prevalence of 13% in the United Kingdom [1]. Ureteric colic typically affects adults of working age [2], with resultant personal and societal cost due to working days missed. There is limited evidence on the impact of stone disease on patients’ quality of life but the severity of the pain is well documented [3].

Most ureteric stones pass spontaneously with conservative or supportive care, such as increased fluid intake and pain relief [4]. However, between a fifth and a third of cases require active intervention [5] because of continuing pain, infection or obstruction to urine drainage. The two standard active intervention options are shock wave lithotripsy (SWL) and ureteroscopic stone treatment (ureteroscopy [URS]). In some cases, a temporary procedure, such as ureteric stent placement or nephrostomy, is needed to treat concurrent infection or obstruction before intervention can safely take place. The use of URS has increased in the last decade [1] although it remains unclear whether the increase is mainly for stones in the ureter, the kidney or both. This change has occurred despite the lack of evidence of clinical or economic benefit of URS to patients or the healthcare system. Studies have suggested that URS is more clinically effective at making patients stone‐free, albeit with a higher complication rate and longer hospital stay [6, 7], but SWL is likely to be more cost‐effective. However, there is marked uncertainty about which treatment pathway is more effective and efficient from the perspective of both the healthcare system (UK NHS) and patients.

We aimed to assess the cost‐effectiveness, resource use implications, quality‐adjusted life‐years (QALYs) and the cost per QALY of SWL compared to URS.

## Methods

An economic evaluation was conducted alongside the Therapeutic Interventions for Stones of the Ureter (TISU) randomized controlled trial (RCT), the design details of which can be found in Dasgupta et al. [8]. In summary, TISU was a non‐inferiority multicentre study, conducted across 25 NHS hospitals, comparing SWL with URS as first‐line active intervention. We recruited patients aged ≥16 years with a diagnosis of a unilateral ureteric stone confirmed by non‐contrast CT. The primary clinical outcome was whether further intervention was required to clear the stone and the primary economic outcome was the incremental cost per QALY. We followed the reference case of the National Institute for Health and Care Excellence (NICE) [9]. We adopted the perspective of the NHS although some personal resource data were collected from the participants. We did not use discounting because participants were followed up for 6 months. We used the cost year 2017/2018 and the currency used was pounds sterling (£).

### Resource Use Data Collection

We estimated resource use and costs for each participant. We considered the resources used for the care pathways that participants followed. We collected information on initial interventions and consequent use of primary and secondary NHS services received by participants using case report forms completed by study site staff for each participant at the time of treatment and up to 6 months post randomization. Data on visits to a GP, prescriptions, over‐the‐counter medications and private healthcare visits were collected via patient‐completed questionnaire at 6 months post randomization.

### NHS and Participant Costs

Details of unit costs were based on published sources, namely, the British National Formulary [10], the NHS reference cost [11] and the Personal Social Services Research Unit costs [12]. The costs of the initial treatment, either SWL or URS, were identified by mapping the reimbursement code provided from the Office of Population Census and Surveys Classification of Invention and Procedures version 4.8. The costs were based on the weighted average reported health‐related group (HRG) activity, which excluded excess bed days. Table 1 provides the codes of the HRGs and the costs we used. Ureteric stents received during the initial URS intervention were not costed as the procedure cost includes stenting. Each care pathway cost also included any inpatient stay that the participants required for complications from the treatment of their ureteric stone. The trim point for inpatient stay for SWL intervention is 1 day and for URS it is 2 days. We used the elective excess bed days cost of URS for any inpatient stay that was greater than the trim point number of days for the procedure (as there is no excess bed days cost for SWL). The inpatient cost for participants who did not receive any intervention was based on the URS HRG cost of non‐elective inpatient excess days.

**Table 1:** Average unit costs of resources.

Resource	Unit cost	Notes [reference]
Medical expulsion therapy (tamsulosin)	£5	Based on a 2‐week dose BNF [10]
GP doctor consultation	£31	Per surgery consultation lasting 9.22 min [12]
GP nurse consultation	£11	Per surgery consultation lasting 15.5 min [12]
X‐ray	£31	Direct access plain film [11]
CT scan	£97	Weighted average cost of imaging: Outpatient CT scans RD20AZ‐RD28Z [11]
Ultrasonography scan	£58	Weighted average cost of imaging: Outpatient ultrasonography scans RD40AZ‐RD46Z [11]
Contrast fluoroscopy	£155	Weighted average cost of imaging: Outpatient contrast procedures RD30AZ‐RD35Z [11]
Night in hospital	£370	Weighted average cost of elective inpatient excess days for LB65 C‐E (Dept. of Health)
	£386	Weighted average cost of non‐elective inpatient excess days for LB65C‐E [11]
Percutaneous insertion of nephrostomy tube M13	£1027	Average cost of unilateral, percutaneous insertion of ureteric stent or nephrostomy YL11Z [11]
Antegrade insertion of stent into ureter M33	£1054	Average cost of intermediate endoscopic ureter procedures, 19 years and over, LB09D [11]
Therapeutic ureteroscopic operations M27	£2123	Weighted average cost of major endoscopic ureter procedures kidney or ureter procedures, 19 years and over, LB65C‐E [11]
Insertion/removal of stent into ureter M29	£1054	Average cost of intermediate endoscopic ureter procedures, 19 years and over, LB09D [11]
SWL M31	£491	Average cost of day case extracorporeal SWL procedures (LB36Z) [11]
Outpatient visit	£110	Average cost of an outpatient visit to urology department (weighted consultant and non‐consultant led), service code 101 [11]

BNF, British National Formulary; GP, general practice; SWL, shockwave lithotripsy.

Participant costs were self‐reported and comprised prescription, over‐the‐counter medication purchases and private visit costs. Estimates of resource utilization were multiplied by unit costs to derive total costs for each item of resource use. We summed these costs to produce a total cost and an average total cost per participant in each care pathway group.

### Quality of Life

We used the EQ‐5D‐3L questionnaire [13] to measure the generic health‐related quality of life/health status; this was completed at baseline (after informed consent but before randomization), directly prior to treatment (pre intervention) and 1 week after intervention/treatment and at 8 weeks and 6 months post randomization. Area under the curve (AUC) was used to estimate QALYs (quality of life multiplied by duration of trial). Calculation of the AUC considered the length of time the patient waited for treatment. Calculation of QALYs for those who were missing a treatment date was based on the post‐randomization time points.

We also used responses from the SF‐12 questionnaire [14] collected at baseline, 8 weeks and 6 months post randomization to estimate QALYs. We mapped these onto the existing SF‐6D measure using the algorithm described by Brazier et al. [15] to allow utility values to be estimated for each time point. These utility scores were transformed into QALYs using the methods described above to provide an alternative measure of QALYs.

### Data Analysis

The economic analysis was based on the intention‐to‐treat principle. All components of costs were described with the appropriate descriptive statistics: mean and sd for continuous and count outcomes. All analyses were conducted using stata® version 15 software (StataCorp LP, College Station, TX, USA). We investigated skewed cost data (due to a small proportion of participants incurring very high costs), using generalized linear models to test alternative model specifications for appropriate fit to the data. These generalized linear models allow for heteroscedasticity by specifying a distributional family which reflects the relationship between mean and variance [16].

We used a modified Park's test which identified Gaussian family as the most appropriate distribution; this allows skewness and assumes that the variance is proportional to the square of the mean. We identified a log as the best link function to specify the relationship between the set of regressors and the conditional mean. The mean incremental QALYs were estimated using ordinary least squares, adjusted for minimization variables (stone size: ≤10 mm or >10 mm; stone location: upper, middle or lower ureter) and baseline EQ‐5D‐3L score. Analysis models were run to estimate the incremental effect of treatment group on costs and QALYs. The coefficient for treatment in the respective models was taken as the estimate of incremental costs for use in the economic evaluation [16, 17].

### Missing Data

Missing data are common for either one or both outcome variables, i.e., the cost and the utility measures in cost‐effectiveness analyses [18]. The patient characteristics of those who had complete data were comparable to those with incomplete data and the data were missing at random. Because more than 10% of complete data were missing, imputation was used for the base‐case analysis [19]. Multivariate imputation by chained equations [20] was used to impute values for missing data. All imputation models included variables for indicators such as treatment allocation and patient characteristics: stone size, stone location (upper, middle and lower ureter), sex and age.

### Incremental Cost‐Effectiveness

Our base‐case analysis was structured on imputed data and sensitivity analysis was performed on the complete case (those with both complete cost and complete QALY) data. Overall results of the cost−utility analysis are reported as incremental cost per QALY gained for care pathways starting with SWL vs URS. We present point estimates of mean costs, QALYs, and incremental cost per QALY of each treatment care pathway. We used non‐parametric bootstrapping of the imputed regression models to consider the impact of sampling uncertainty to generate a probability of cost‐effectiveness at several threshold values of decision‐makers willingness to pay (WTP) for a QALY gain. Non‐parametric boot strapping methods were used to estimate 95% CIs for treatment effects on costs and QALYs, to summarize the uncertainty surrounding the incremental cost‐effectiveness ratios (ICERS). Incremental cost‐effectiveness results are presented in terms of cost‐effectiveness acceptability curves (CEACs). This presentation allows visual representation of the joint uncertainty in the effect sizes for cost and QALY estimates, illustrating the probability of the specified intervention (in this case SWL) falling into each of the following quadrants of the cost‐effectiveness plane: (i) less costly and more effective; (ii) more costly and less effective; (iii) less costly and less effective; or (iv) more costly and more effective.

The CEACs were generated using these 1000 estimates, using the net monetary benefit (NMB) approach. The NMB associated with a given treatment option is given by the formula:

$$\mathrm{NMB}=\left(\text{Effect}\times \mathrm{Rc}\right)-\text{cost},$$

where Effects are measured in QALYs and Rc is the ceiling ratio of WTP per QALY.

Using this formula, the strategy with the greatest NMB is identified for each of the 1000 bootstrapped replicates of the analysis, for different ceiling ratios of WTP per QALY. For the purposes of the base‐case analysis, Rc was set at £30 000, the upper end of the commonly accepted range of ICERs considered to offer good value for money by NICE [9]. A number of alternative threshold values were presented within the table.

### Sensitivity Analysis

We used deterministic sensitivity analysis to explore the impact and importance of the following assumptions, uncertainties and analysis models on the cost‐effectiveness findings. We used the complete case (for participants with both cost and QALY data) to assess the impact of missing data on the results. There is some uncertainty as to whether the dimensions in the EQ‐5D‐3L are sensitive to capture the loss in quality of life, particularly, in reference to acute pain. Therefore, SF‐12 responses were mapped on the SF‐6D measure using the algorithm described by Brazier et al. [15] to facilitate the estimation of utility values for each time point. Analysis based on the assumption that all patients with missing EQ‐5D‐3L at 6 months were in full health because the stone had passed was conducted. The NHS reference unit cost for the HRG for SWL is almost a quarter of the cost of URS. Several studies outside an NHS setting [21] have indicated that SWL costs more than URS. Therefore, sensitivity analyses were undertaken using the elective inpatient tariff of SWL.

## Results

In total, 613 participants were recruited from 25 centres: 306 were randomized to the SWL care pathway arm and 307 to the URS care pathway arm (of which 303 and 306, respectively, were included in the study). The clinical effectiveness results are reported elsewhere [8]. In summary, the groups were well balanced at baseline. The mean (sd) age was 51 (14) years and the majority of participants (80%) were men. With regard to stone size, 95% of participants had a stone of less than or equal to 10 mm, and 45% of stones were in the upper ureter and 38% in the lower ureter. The clinical analysis found that the further intervention rate at 6 months in the SWL arm was 11.7% (95% CI 5.6–17.8%) higher than that in the URS arm, but this difference was non‐inferior.

### Resource Use

Details of resource use can be found in the resource‐use table in the supporting information (Table S1). Resource use was higher for the participants allocated to the SWL care pathway for outpatient hospital visits, for all imaging apart from intravenous urogram, and for SWL. It is common for a stent to be inserted during the URS procedure; therefore, stents were not reported as additional resource use if they were inserted when participants received URS, but they were included as resource use and costed when they were removed. The URS arm had higher resource use for URS and stent removals. Thirty‐two participants reported purchasing over‐the‐counter medicines: 12 in the SWL group and 20 in the URS group. Two participants reported that they saw a private healthcare provider, one in each group.

### Costs

Table 2 provides information on the mean cost per participant according to category of resource use. Costs were higher for the SWL pathway for hospital visits, all imaging apart from intravenous urogram, endoscopic stent insertion and SWL. Endoscopic stent insertion costs were higher for the SWL pathway because the SWL unit cost does not include stenting but if a stent was inserted as part of/at the same time as a URS procedure then we did not cost it in addition to the cost of the URS procedure as this is included in the unit cost for URS. However, the difference in cost was minimal. Costs in the URS pathway for URS and stent removals were statistically significantly higher. The total complete‐case analysis costs were higher for the URS pathway, which was mainly driven by the cost of URS treatment.

**Table 2:** Mean resource use costs and quality‐of‐life measures.

Resource	SWL			URS			Difference SWL vs URS^*^	
Costs (£)	*N*	Mean	sd	*N*	Mean	sd	Mean	95% CI
Medical expulsion therapy	191	1.18	2.10	191	1.13	2.06	0.02	−0.23 to 0.28
GP consultation	189	8.22	24.83	191	7.75	29.60	0.86	−3.65 to 5.38
General practice nurse consultation	191	1.84	11.02	192	0.92	4.41	0.69	−0.36 to 1.74
Outpatient hospital visits	303	173.67	104.10	302	92.01	96.12	80.94	57.71 to 104.17
X‐ray	303	48.16	35.35	303	21.52	26.25	26.21	20.41 to 31.99
Ultrasonography	303	21.88	45.83	303	4.60	18.95	17.74	−2.83 to 38.31
CT scan	303	26.03	51.50	303	18.45	45.83	8.20	1.57 to 14.83
Intravenous urogram	303	0.20	2.42	303	0.10	1.71	0.12	−0.31 to 0.55
Nephrostomy tube	303	6.43	78.97	303	3.21	55.93	3.47	−11.42 to 18.37
Antegrade stent insert/removal	303	24.10	178.46	303	6.89	84.61	17.48	−8.21 to 43.17
URS	303	633.89	1030.48	303	1894.54	997.19	−1282.18	−1468.92 to −1095.43
Ureteric stent insertion	303	10.33	103.45	306	3.41	59.63	−6.50	−10.40 to 23.41
Ureteric stent removal	303	165.25	450.79	303	333.95	536.72	−173.63	−256.59 to −90.66
SWL	303	506.77	399.64	303	49.19	200.30	458.24	371.92 to 544.56
Inpatient stay (days)	298	160.69	453.39	290	138.83	442.36	4.46	−50.23 to 59.15
Total cost^†^	182	1549.53	1586.10	179	2498.33	1436.43	−808.20	−1044.24 to −571.00
**Quality‐of‐life measures**								
EQ‐5D‐3L								
Baseline	298	0.737	0.263	297	0.729	0.303		
Pre‐treatment	252	0.735	0.260	211	0.758	0.272	−0.041	−0.085 to 0.002
1 week post treatment	186	0.756	0.267	175	0.757	0.263	−0.007	−0.068 to 0.055
8 weeks post randomization	149	0.797	0.293	152	0.874	0.207	−0.081	−0.152 to −0.009
6 months post randomization	130	0.837	0.289	143	0.912	0.182	−0.081	−0.146 to −0.016
QALY^‡^	70	0.407	0.116	74	0.436	0.070	−0.029	−0.062 to 0.005
EQ‐5D VAS								
Baseline	282	68	24	283	67	27		
Pre‐treatment	235	69	25	198	74	22	−4	−7 to −1
1 week post treatment	180	74	22	172	74	20	−1	−7 to 5
8 weeks post randomization	150	77	21	153	79	21	−4	−9 to 1
6 months post randomization	131	78	21	143	81	18	−3	−9 to 3
SF‐6D								
Baseline	195	0.699	0.168	193	0.737	0.175		
8 weeks post randomization	106	0.762	0.169	107	0.782	0.151	−0.003	−0.047 to 0.040
6 months post randomization	104	0.789	0.173	109	0.837	0.139	−0.069	−0.123 to −0.015
QALY^‡^	45	0.393	0.075	51	0.400	0.064	−0.009	−0.036 to 0.018

^*^ Differences based on regression model adjusting for baseline EQ‐5D‐3L and minimization variables (trial centre [site], stone size [≤10 mm or >10 mm] and stone location [upper, middle and lower ureter]) age and gender.

^†^ Total costs are based on the total resource use that the participants reported over the 6‐month period. On average, the SWL pathway cost ~£800 less than URS.

^‡^ The maximum the QALY value can be is 0.5 as it is measured over a 6‐month period. QALYs were calculated for each participant using data from all the different time points. Most participants were missing data for at least one time point. Of the participants who had all EQ‐5D‐3L data, those in the SWL pathway had ~0.030 QALYs fewer than those in the URS pathway.

EQ‐5D VAS, EuroQol visual analogue scale; GP, general practitioner; *n*, number of responses; QALY, quality‐adjusted life‐year; SWL, shock wave lithotripsy; URS, ureteroscopy.

The SWL group spent, on average, £2 and URS spent £3 on over‐the‐counter medicine purchases. The mean cost spent on private care was £24 for the SWL group and £2 for the URS group.

### Quality‐Adjusted Life‐Years

Table 2 shows the EQ‐5D‐3L, visual analogue scale (VAS) and SF‐6D utility scores for each care pathway at different time points. The baseline utility scores were similar. The EQ‐5D‐3L utility scores at pre‐treatment, 8 weeks post randomization and 6 months post randomization were higher for URS than for SWL. The estimated mean QALYS were 0.411 (0.112) for the SWL pathway and 0.439 (0.070) for the URS pathway. The adjusted mean QALY difference for the SWL care pathway was −0.032 (95% CI −0.061, −0.058). The VAS scores were higher in the URS group at each time point, but differences were small. The mean (sd) estimated QALYs for SF‐6D utility scores were 0.393 (0.075) for the SWL group and 0.400 (0.064) for the URS group. The adjusted mean QALY difference (SWL vs URS) was −0.014 (95% CI −0.043, 0.010). The complete‐case QALY results should be interpreted with caution considering the high proportion of missing data.

### Cost‐Effectiveness Analysis

**Fig. 1:**
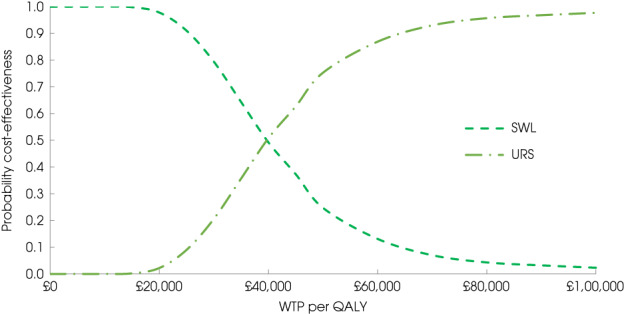
Cost‐effectiveness acceptability curve for shockwave lithotripsy (SWL) vs ureteroscopy (URS): base‐case analysis based on multiple imputation data. The probability that either treatment will be cost‐effective (*y*‐axis) depends on society's willingness to pay (WTP) for an additional quality‐adjusted life‐year (QALY; *x*‐axis). The threshold in the UK is between £20 000 and £30 000. For example, there is a 79% (98%) probability that SWL is cost‐effective at the £30 000 (£20 000) WTP threshold.

The results of the base‐case analysis are reported in Table 3. The base‐case analysis (using multiple imputation) showed that the mean cost for participants on the SWL care pathway was £809 (95% CI −1061 to −551) less than for those on the URS pathway but the participants had 0.021 (95% CI −0.033 to −0.010) fewer QALYs than participants on the URS pathway. The point estimate of the incremental cost per QALY was £39 118 cost saving per QALY lost and the uncertainty around this estimate is illustrated in Fig. 1 and Fig. S1. This means that a decision‐maker would save £39 118 for each lost QALY with 79% probability that SWL would be considered cost‐effective at a society's WTP for 1 QALY threshold of £30 000.

**Table 3:** Incremental cost‐effectiveness from an NHS perspective.

	Cost	Difference^*^	QALY	Difference	ICER	Probability of being cost‐effective at different WTP thresholds (%)^†^				
	£	£			£/QALY	0	10 000	20 000	30 000	50 000
**Base‐case imputed data analysis**										
SWL	1790		0.403			1	1	0.98	0.79	0.25
URS	2599	−809^‡^	0.424	−0.021	39 118	0	0	0.02	0.21	0.75
**Complete‐case analysis**										
SWL	1584		0.407			1	1	0.96	0.80	0.46
URS	2932	−1348	0.436	−0.029	46 297	0	0	0.04	0.20	0.52
**Imputed data using SF‐6D utility scores**										
SWL	1790		0.385			1	1	1	1	1
URS	2599	−809	0.387	−0.002	432 432	0	0	0	0	0
**Complete‐case using SF‐6D utility scores**										
SWL	2102		0.388			0.81	0.76	0.70	0.64	0.53
URS	2502	−500	0.398	−0.010	52 313	0.20	0.24	0.30	0.36	0.47
**Assuming all patients with missing EQ‐5D‐3L 6‐month scores were in full health at 6 months as stones had passed**										
SWL	1790		0.423			1	1	1	0.95	0.65
URS	2599	−809	0.437	−0.014	57 889	0	0	0	0.05	035
**Higher cost of SWL assuming 25% of patients were inpatients**										
SWL	1952		0.403			1	1	0.90	0.58	0.13
URS	2614	−663	0.424	−0.021	32 034	0	0	0.10	0.42	0.87
**Higher cost of SWL assuming 50% of patients were inpatients**										
SWL	2073		0.403			1	0.98	0.77	0.42	0.08
URS	2627	−555	0.424	−0.021	26 820	0	0.02	0.23	0.58	0.92
**Higher cost of SWL assuming 75% of patients were inpatients**										
SWL	2190		0.403			1	0.94	0.60	0.23	0.04
URS	2642	−453	0.424	−0.021	21 888	0	0.06	0.40	0.77	0.96
**Higher cost of SWL assuming 100% of patients were inpatients**										
SWL	2306		0.403			0.99	0.81	0.38	0.13	0.02
URS	2652	−346	0.424	−0.021	16 710	0.01	0.19	0.62	0.87	0.98
**Higher cost of SWL assuming 100% of patients were inpatients, complete case**										
SWL	2142		0.407			1	0.95	0.73	0.51	0.25
URS	2961	−818	0.435	−0.028	29 434	0	0.05	0.27	0.49	0.75

^*^ Differences based on generalized linear model adjusting for baseline EQ‐5D‐3L and minimization variables (trial centre [site], stone size [≤10 mm or >10 mm] and stone location [upper, middle and lower ureter] age and gender).

^†^ The probability that either treatment will be cost‐effective depends on society's WTP for an additional QALY. The WTP threshold in the UK is £20 000–30 000. There is a 98% and 79% probability that SWL is cost effective at the £20 000 and £30 000 WTP threshold, respectively. SWL would therefore be considered cost‐effective at the NICE recommended threshold of £20 000–30 000 per QALY.

^‡^ SWL costs less and is less effective than URS.

ICER, incremental cost‐effectiveness ratio; NICE, National Institute of Health and Care Excellence; QALY, quality‐adjusted life‐year; SWL, shock wave lithotripsy; URS, ureteroscopy; WTP, willingness to pay.

Figure S1 shows that many bootstrapped iterations lie to the left of the vertical and below the horizontal axes, indicating that the SWL is less costly when compared to URS, with marginally fewer QALYs achieved through the intervention.

### Sensitivity Analysis

The results of the sensitivity analyses (Table 3) were the same in direction to those of the base‐case analysis: SWL cost less, with values ranging between £346 (assuming all SWLs were performed as an inpatient procedure) and £1348 (for the complete‐case analysis). The QALY results were in the same direction also: SWL was less effective and the QALY values ranged from 0.002 (based on imputed SF‐12 scores) to 0.029 (complete‐case analysis). The probability that society will be willing to pay for a QALY loss was between 13% to 100% for the £30 000 WTP threshold.

## Discussion

This is the first economic evaluation that has directly compared clinical pathways starting with SWL or URS in an RCT setting. The results suggest that participants in the SWL arm cost the NHS less (average £809) but have fewer QALYs (0.021) than participants in the URS arm. The difference in cost is mainly driven by the unit intervention cost of URS of £2123 [11], which is on average four times as high as the unit cost of SWL (£491). Additionally, more participants in the URS pathway received a stent following fragmentation of the stone. The unit cost of stent insertion or removal procedure (£1027) is twice that of SWL. Endoscopic stent insertion during the URS procedure was not costed separately as it was included in the overall cost of the URS. Although the participants that followed the SWL pathway subsequently used more resources, the additional costs were not enough to offset the initial high cost of URS and stent removal costs in the URS care pathway. The ICER per QALY lost in the SWL pathway is £39 118 and there is a 79% and 98% probability (at £30 000 and £20 000 WTP for a QALY threshold) that the SWL pathway would be considered cost‐effective in a similar healthcare setting.

The direction of difference in utility scores derived from both the EQ‐5D‐3L and SF‐12 was the same, however, the magnitude of the difference was higher for the EQ‐5D‐3L scores than SF‐12 scores. On average SWL was associated with lower QALYs gained than URS and the QALY difference in the EQ‐5D‐3L scores was statistically significant. Quality‐of‐life data were collected several times after randomization to capture any changes in quality of life and the calculation of the QALY took into account the time the participants waited for the appointment. The difference in QALY (0.021) translates into 10 more days in perfect for the URS group over the 6‐month follow‐up period. Although the imputation analysis indicated that this was statistically significant, no analysis was conducted to establish whether this was a minimal important difference to the patient. The QALY difference score obtained using the SF‐6D was not statistically significant, but this should be interpreted taking into account that there were more missing SF‐12 data. Overall, participant's utility scores increased over the follow‐up time which reflects their pathway back to better health. Analysis was conducted based on the assumption that all participants that were missing EQ‐5D‐3L data had passed their stone at 6 months and the results were similar to the base‐case analysis.

The base‐case analysis ICER results based on the imputed data indicated that SWL had a 79% chance of being considered cost‐effective at the £30 000 WTP threshold, while the sensitivity analysis using complete‐case data indicated that SWL had an 80% chance of being considered cost‐effective at the same threshold. Similar results were noted in the SF‐6D score sensitivity analysis that explored the effect of the higher cost of SWL. However, the results of varying the unit cost of SWL by assuming that a greater proportion of those in the SWL arm would have an inpatient elective SWL procedure suggested that the higher the unit cost of SWL, the lower the probability that SWL would be considered cost‐effective at the different WTP thresholds. The ICER results suggest that, although SWL costs less than URS and was less effective than URS, they were sensitive to the assumptions made as they ranged from £16 710 to £432 432. The probability that SWL would be considered cost‐effective at the £30 000 WTP threshold ranged from 13%, when a higher cost of SWL was assumed, to 100% when the analysis was based on the SF‐6D utility scores.

The cost of both procedures was taken from the NHS reference costs and the cost of SWL was assumed to refer to each session. HRG costing is based on both top‐down costing, whereby cost pools (used to collect indirect and overhead costs) are allocated to HRGs using the total cost of that cost pool weighted for each HRG based on the best available data, and bottom‐up costing, which builds up the costs of an HRG from the bottom up where the actual costs are known, for example, prosthetics in hip replacement HRGs [22]. There are issues around using the NHS reference cost for SWL, as although it is an average, it is likely to be skewed towards a lower cost by the high‐volume centres. Similarly, if SWL was more available then machines only being used for small volumes could drive up the NHS reference cost. One of the cost efficiencies of SWL is its functioning as an outpatient intervention, and thus the incremental expenditure if counting over a certain volume of these cases as inpatients may reduce this efficiency. Therefore, sensitivity analyses were conducted on the SWL cost.

The cost‐effectiveness results of the TISU study corroborate findings of studies [23, 24] that performed SWL as primary treatment for stones between 10 and 20 mm. SWL was undertaken in an outpatient setting and patients did not receive sedation or anaesthesia but had analgesia according to their tolerance. Flexible URS was typically performed under general anaesthesia. The authors concluded that SWL on average is cheaper than URS. Similar results were reported by Koo et al. [24] in their UK study comparing the SWL and flexible ureteroscopic laser lithotripsy procedure, albeit in the different setting of elective renal stone disease, rather than acute ureteric stone disease.

The authors of a systematic review [21] reported that URS was more cost‐effective than SWL for stone treatment. However, the measurement of cost varied across studies and the cost of procedures also varied among healthcare systems. The review was based on retrospective case series and the authors indicated that the evidence base was poor and there was a need for large RCTs. Lotan et al. [25] reported that URS was the most cost‐effective treatment strategy for ureteric stones at all locations after observation had failed. They cited the high cost of purchasing and maintaining a lithotriptor as the driver of the higher SWL cost in their study. Pearle et al. [26] also reported that SWL was slightly more costly than URS and Cone et al. [27] reported that URS had superior clinical and cost‐effectiveness over SWL.

The results and conclusions of these studies are similar to those reported by Constanti et al. [28], who compared the total cost of a treatment strategy starting with URS vs a strategy starting with extracorporeal SWL. The study was model‐based and used data that had been collected to inform the NICE guideline NG118 [29] on the costing analysis of surgical treatments of renal and ureteric stones. Their results showed it would cost >£2000 more per patient to clear a stone with URS than with extracorporeal SWL. Their QALYs were based on exploratory calculations as no studies had collected QALY data. The QALY threshold analysis and exploratory QALY work found that the QALY difference would have to be very high for URS to be cost‐effective, and the quality‐of‐life difference needed between a stone‐free and non‐stone‐free person to make URS cost‐effective was unfeasible. They concluded that URS was unlikely to be cost‐effective for ureteric stones of <10 mm, and extracorporeal SWL should be the first‐line treatment, as it was found to have a better balance of costs and benefits.

One of the limitations of our study was the low return and completion rates of the patient questionnaires, which led to missing data. This was addressed by imputing the missing data. Comparison of participant data indicated that there were no differences in the characteristics of those with missing and those without missing data. On average, the SWL arm cost less than the URS arm and was associated with fewer QALYs gained than URS. However, the complete‐case data favoured SWL, with an ICER of £46 297 cost saving per reduction in QALY as well as a similar chance (80% vs 79%) of being considered cost‐effective at the £30 000 WTP threshold or 96% vs 98% at the £20 000 threshold.

Another challenge in this study was the valuation of resource use for the two interventions. The costs of SWL and URS were based on HRG tariffs, which are traditionally based on the average cost of services reported by the NHS providers. HRGs provide a currency payment for the average patient. Ideally a bottom‐up method should have been applied to identify, measure and value resource use rather than the use tariff‐based costs. This was not practical in this study and the HRG tariffs were considered as appropriate costs, and sensitivity analyses were conducted to measure the impact of varying the costs of SWL.

One of the strengths of this study is that it is the first study, to our knowledge, that has measured and reported quality of life using generic tools in order to compare the two interventions SWL and URS [15]. The data were collected at several time points to capture changes that participants experienced as their symptoms resolved. Overall, quality of life for participants in both the SWL and URS care pathway groups increased, and at 8‐week and 6‐month follow‐ups the utility scores based on the EQ‐5D‐3L instrument were statistically significantly higher for URS when calculating the AUC. The QALY difference translated into approximately 10 more healthy days over the 6‐month period for the patients in the URS care pathway.

In conclusion, we found that the SWL pathway was cheaper than URS, and the subsequent intervention costs were unlikely to exceed those incurred by use of URS. However, SWL results in fewer QALYs gained. The ICER was approximately £40 000 saved for each QALY lost and the probability that the SWL pathway is cost‐effective was 79% at the WTP threshold of £30 000 and 98% at the £20 000 WTP threshold; therefore, decision‐makers need to determine whether costs saved justify the loss of QALYs.

## Disclosure of Interests

None of the authors had any conflicts of interest to disclose.

**Abbreviations**
AUC: area under the curve
CEAC: cost‐effectiveness acceptability curve
HRG: health‐related group
ICER: incremental cost‐effectiveness ratio
NICE: National Institute for Health and Care Excellence
NMB: net monetary benefit
QALY: quality‐adjusted life‐year
RCT: randomized controlled trial
SWL: shock wave lithotripsy
TISU: Therapeutic Interventions for Stones of the Ureter
URS: ureteroscopy
WTP: willingness to pay

## Supporting information


**Fig. S1.** Scatter plot of incremental cost and incremental QALYs (imputed data): shockwave lithotripsy (SWL) vs ureteroscopy (URS).

**Table S1.** Average resource use.
