# Supplementary material for: Cost−utility analysis of shockwave lithotripsy vs ureteroscopic stone treatment in adults
Source: BJU Int. 2022 Aug 16;131(2):253–61. doi: 10.1111/bju.15862 (PMC10087721; doi:10.1111/bju.15862)
Supplement: Supplementary file 1 — Fig. S1. Scatter plot of incremental cost and incremental QALYs (imputed data): shockwave lithotripsy (SWL) vs ureteroscopy (URS). [file BJU-131-253-s001.docx]

**Figure S1: Scatter plot of incremental cost and incremental QALYs (imputed data): Shockwave lithotripsy (SWL) versus Ureteroscopy (URS)**

QALYs quality adjusted life years. The incremental (difference) values in the cost effectiveness plane show that on average SWL costs less than URS and it has lower QALYs than URS.
