# Supplementary material for: Cost−utility analysis of shockwave lithotripsy vs ureteroscopic stone treatment in adults
Source: BJU Int. 2022 Aug 16;131(2):253–61. doi: 10.1111/bju.15862 (PMC10087721; doi:10.1111/bju.15862)
Supplement: Supplementary file 2 — Table S1. Average resource use. [file BJU-131-253-s002.docx]

**Table S1 Average resource use**

| **Resource** | **Shockwave lithotripsy (SWL) N=303** | | | **Ureteroscopy (URS) N=306** | | | **Difference SWL vs URS*** | |
| --- | --- | --- | --- | --- | --- | --- | --- | --- |
|  | n | Mean | sd | n | Mean | sd | Mean | 95% CI |
| Medical expulsion therapy | 191 | 0.24 | 0.43 | 191 | 0.23 | 0.42 | 0.20 | -0.60 to 1.00 |
| GP Doctor visits | 191 | 0.17 | 1.00 | 192 | 0.08 | 0.40 | 0.02 | -0.10 to 0.15 |
| GP Nurse visits | 189 | 0.22 | 0.67 | 191 | 0.21 | 0.80 | 0.06 | -0.03 to 0.16 |
| Outpatient hospital visits | 303 | 1.59 | 0.95 | 302 | 0.84 | 0.88 | 0.74 | 0.53 to 0.95 |
| X-ray | 303 | 1.62 | 1.19 | 303 | 0.72 | 0.88 | 0.88 | 0.69 to 1.07 |
| Ultra-sound | 303 | 0.39 | 0.82 | 303 | 0.08 | 0.34 | 0.32 | -0.05 to 0.69 |
| Computerised tomography scan | 303 | 0.26 | 0.52 | 303 | 0.18 | 0.46 | 0.08 | 0.02 to 0.15 |
| Intravenous urogram | 303 | 0.01 | 0.08 | 303 | 0.00 | 0.06 | 0.00 | -0.01 to 0.02 |
| Nephrostomy tube | 303 | 0.01 | 0.08 | 303 | 0.00 | 0.06 | 0.00 | -0.01 to 0.02 |
| Antegrade stent insertion | 303 | 0.02 | 0.17 | 303 | 0.01 | 0.08 | 0.02 | -0.01 to 0.04 |
| Ureteroscopy | 303 | 0.29 | 0.48 | 303 | 0.88 | 0.46 | 0.59 | -0.68 to -0.51 |
| Endoscopic stent insertion | 303 | 0.01 | 0.10 | 303 | 0.00 | 0.06 | 0.01 | -0.01 to 0.02 |
| Endoscopic stent removal | 303 | 0.16 | 0.43 | 303 | 0.32 | 0.51 | 0.17 | -0.25 to -0.09 |
| Shock wave lithotripsy | 303 | 1.12 | 0.88 | 303 | 0.11 | 0.44 | 0.01 | -0.16 to 0.19 |
| Inpatient stay (Days) | 298 | 0.53 | 1.49 | 290 | 0.46 | 1.45 | 0.20 | -0.60 to 1.00 |

*Differences based on regression model adjusting for baseline EQ-5D-3L and minimisation variables (trial centre (site), stone size (≤ 10 mm or ˃10 mm) and stone location (upper, middle and lower ureter; factors) age and gender. SD standard deviation n sample size CI confidence interval. Endoscopic stent insertion was only included as response use when it was performed as a single procedure.
